# Multi-Attention Segmentation Networks Combined with the Sobel Operator for Medical Images

**DOI:** 10.3390/s23052546

**Published:** 2023-02-24

**Authors:** Fangfang Lu, Chi Tang, Tianxiang Liu, Zhihao Zhang, Leida Li

**Affiliations:** 1 College of Computer Science and Technology, Shanghai University of Electric Power, Shanghai 201399, China; 2 Department of Electronic Engineering, School of Electronic Information and Electrical Engineering, Shanghai Jiao Tong University, Shanghai 200240, China; 3 School of Artificial Intelligence, Xidian University, Xi’an 710000, China

**Keywords:** lesion segmentation, deep learning, COVID-19, multi-attention

## Abstract

Medical images are used as an important basis for diagnosing diseases, among which CT images are seen as an important tool for diagnosing lung lesions. However, manual segmentation of infected areas in CT images is time-consuming and laborious. With its excellent feature extraction capabilities, a deep learning-based method has been widely used for automatic lesion segmentation of COVID-19 CT images. However, the segmentation accuracy of these methods is still limited. To effectively quantify the severity of lung infections, we propose a Sobel operator combined with multi-attention networks for COVID-19 lesion segmentation (SMA-Net). In our SMA-Net method, an edge feature fusion module uses the Sobel operator to add edge detail information to the input image. To guide the network to focus on key regions, SMA-Net introduces a self-attentive channel attention mechanism and a spatial linear attention mechanism. In addition, the Tversky loss function is adopted for the segmentation network for small lesions. Comparative experiments on COVID-19 public datasets show that the average Dice similarity coefficient (DSC) and joint intersection over union (IOU) of the proposed SMA-Net model are 86.1% and 77.8%, respectively, which are better than those in most existing segmentation networks.

## 1. Introduction

Coronavirus disease 2019 (COVID-19) is an epidemic disease caused by a new coronavirus (formerly known as 2019 nCoV). This new coronavirus has strong adaptability, as thus far, it has produced 11 different mutant strains. According to the latest statistics from the Johns Hopkins Center for Systems Science and Engineering (CSSE) (updated 8 October 2022), the number of confirmed COVID-19 cases worldwide has reached 621 million, including 6.56 million deaths. Currently, the reverse transcription-polymerase chain reaction (RT-PCR) is the standard test for diagnosing COVID-19 [1]. However, the RT-PCR test has the possibility of false negatives when the nucleic acid content of the new coronavirus is too low in the test sample. The missed diagnosis cases caused by false negatives will lead to more widespread transmission, which is extremely unfavorable for the prevention and control of the epidemic [2].

In order to better suppress the spread of the coronavirus, chest computed tomography (CT) images have become an important tool for diagnosing COVID-19. The studies in [3,4] showed that CT scans have high sensitivity, and abnormal features such as ground-glass opacity (GGO), consolidation and rare features in CT images can reflect the severity of cases in patients. However, it takes a lot of time to manually segment the lesion areas in CT images, and for an experienced radiologist, it takes about 21.5 min to find the diagnostic results of each case by analyzing CT images [5]. Therefore, it is necessary to propose an automatic lesion segmentation method to assist doctors with diagnoses. Recently, with the powerful feature extraction capability of deep convolutional neural networks, a deep learning-based method has been widely used in medical image processing [6,7]. Wang et al. [8] developed a deep learning method combined with CT classification and segmentation that can extract the CT image features of COVID-19 patients and provide medical diagnoses for doctors. Matteo et al. [9] proposed a lightweight convolutional neural network for distinguishing CT images of COVID-19 patients from healthy CT images.

It is worth noting that the encoder-decoder structure is the most common one used in lesion segmentation models. Many studies [10,11] have confirmed that this structure has good segmentation performance and robustness. As a result, a number of studies have been conducted on the segmentation of COVID-19 lesions by using encoder-decoder structures. An FCN [12], segnet [13], UNet [14] and deeplav3 [15] have been applied to the COVID-19 segmentation task. In addition, UNet and its variants have also been applied to the COVID-19 segmentation task. Chen et al. [16] used UNet combined with a residual network to achieve automatic segmentation of COVID-19 lesions. Bhatia et al. [17] proposed a U-Net++-based segmentation model for identifying 2019-nCoV pneumonia lesions in the chest CT images of patients. Although these methods are more efficient than manual segmentation, they still have shortcomings in segmentation accuracy. They tend to have the following problems. (1) Although the encoder-decoder structure can extract high-level features with rich semantics, it will lose spatial detail information, such as the edge information of the lesion area when the encoder performs downsampling. (2) These networks lack an effective mechanism to learn the channel information and spatial information of features. (3) The previous loss function of semantic segmentation is not suitable for the lesion segmentation task of COVID-19, which makes the network insensitive for small lesion areas.

To solve the above problems, we propose a Sobel operator combined with multi-attention networks (SMA-Net) to segment the lesions of COVID-19. Different from previous methods, we pay more attention to the edge information of images. We propose a self-attentive channel attention mechanism and spatial attention mechanism to guide the network in the concatenation of low-level and high-level features for feature extraction. The Tversky loss function adopted by SMA-Net can take into account the small lesion area and improve its sensitivity.

Our contributions are summarized as follows:

(1) We propose a module for fusing COVID-19 CT images and their edge features to provide more detailed information for the network. This module uses the Sobel edge detection operator to obtain edge information.

(2) We propose a self-attentive channel attention mechanism with a spatial linear attention mechanism module that is independent of the resolution size of the feature map, which we apply to the concatenation of low-level features with high-level features. This enables the network to focus on important semantic information, thereby improving the segmentation performance of the network.

(3) SMA-Net has a suitable loss function for small lesion areas of COVID-19. Compared with other segmentation methods, SMA-Net has better segmentation accuracy in small lesions.

## 2. Related Works

In this section, we briefly review three types of works that are most related to our work, including semantic segmentation of CT images, edge detection and the self-attention mechanism.

### 2.1. Semantic Segmentation of CT Images

Due to the high cost of manual segmentation of medical images, image segmentation methods are widely used for medical image segmentation. We summarize some segmentation methods for medical images and compare their advantages and disadvantages as shown in Table 1:

(1) Superpixel segmentation methods: Due to the difference in texture between the diseased and healthy regions in CT images, segmentation of CT images can be accomplished using superpixel segmentation methods. Di et al. [18] proposed a framework for automatic segmentation of liver tumors using superpixel segmentation combined with support vector machine algorithms. With the development of deep learning, superpixel segmentation methods combined with deep learning can also obtain better segmentation results. Liu et al. [19] used neural networks to extract the depth features from superpixels of CT images of interstitial lung lesions, and the depth features were fed into a random forest classifier to obtain segmentation results.
(2) Watershed segmentation methods: Ajam et al. [20] completed segmentation of the infarcted region of brain CT images of stroke patients using marker-controlled watershed segmentation. Anter et al. [21] used a fast fuzzy C-means clustering algorithm to improve the results of watershed segmentation and achieve the segmentation of tumors in liver CT images.
(3) Active contour methods: Qiang et al. [22] used threshold segmentation and region growth segmentation to isolate lung parenchyma from lung CT images and an active contour segmentation algorithm to segment lung nodules from lung parenchyma.
(4) Deep learning methods: Jonathan et al. proposed the first fully convolutional network (FCN) for semantic segmentation in 2015 [12]. Since then, Olaf Ronneberger et al. proposed UNet [14], a semantic segmentation model dedicated to medical images based on the idea of the FCN. UNet has been widely used in lesion segmentation of COVID-19 CT images with excellent performance. UNet and its variants (UNet++, UNet3+, 3D-UNet, VNet and VBNet) have been a commonly used technique for medical image segmentation, and many COVID-19 studies are based on them. Xie et al. achieved better segmentation of lung lesions using a dual-UNet network [23]. Due to the similarity between COVID-19 and common pneumonia, Yin et al. [24] introduced a compressed attention mechanism and a pyramid pooling module in UNet in order to strengthen the differences and connections between pixels so as to improve the segmentation performance. For small lesions of COVID-19, Owais et al. [25] proposed a dual-scale dilated fusion network to segment small lesion regions. In addition, the choice of loss function also affects the performance of the segmentation model. SRGNet designs a new edge-assisted loss function to enhance the spatial constraints of features [26]. In order to better segment small lesions, Budak et al. introduced focal Tversky loss to improve the contribution of small lesions to the loss [27]. MultiR-Net [28] includes two subnetworks which are used for classification and segmentation tasks of COVID-19. It also proposes a new loss function to enhance the interaction of the two subnetworks. To reduce the dependence on labels, Mohamed et al. [29] applied the few-shot learning (FSL) method to propose a new semi-supervised neocrown pneumonia segmentation framework. Although these methods have good feature extraction capabilities, and these methods often lose some edge information about lesions during downsampling.

**Table 1:** Comparison of the advantages and disadvantages of our method (SMA-Net) with previous methods.

Model	Learning Method	Supervision Method	Lightweight	Encode-Decode	Attention
[18]			×	×	×
[19]	Machine Learning	Unsupervised	×	×	×
[20]			×	×	×
[22]			×	×	×
[12]			×	×	×
[14]	Deep Learning	Supervised	×	*√*	×
[30]			*√*	*√*	*√*
[29]	Deep Learning	Semi-Supervised	×	*√*	×
[31]			×	*√*	*√*
Ours	Deep Learning	Supervised	×	*√*	*√*

### 2.2. Edge Detection

The edge information is the most basic feature of an image. Edge detection is used to extract the edge features of the images. With the development of deep learning, edge detection methods combined with deep learning have been widely proposed. He et al. [32] proposed a bidirectional cascade network for hierarchical supervision of CNNs for efficient layer-specific edge detection. Edge information is a part of semantic segmentation information, and making full use of edge information can improve the performance of a segmentation network. PG et al. [33] combined the segmentation task and the edge detection task and added a side output part in UNet for edge extraction and depth supervision. In the semantic segmentation model of an encoding-decoding configuration, low-level features contain rich edge information. In order to make better use of edge information, Fan et al. [34] used a Laplace operator combined with convolution calculation to extract the binary map of the label edge features. The binary map is used to guide the learning of the low-level features of the encoder. Inf-Net [31] proposed an edge attention module to guide feature extraction which sends low-level features into filters to generate edge maps. To constrain low-level feature extraction, it uses a BCE loss function to measure the dissimilarity between the edge maps and ground truth. Edge information affects the performance of network segmentation. However, the existing lesion segmentation network of CT images of COVID-19 patients is lacking in attention to the edge information. To this end, we will use the Sobel operator to provide rich edge information for the segmentation network to improve the problem of loss of edge information in the network downsampling process.

### 2.3. Self-Attention Mechanism

In order to focus on important information from a large amount of information, the attention mechanism has become an indispensable module of deep learning. It has been widely used in the fields of NLP and CV. Bahdanau et al. [35] first used an attention mechanism for machine translation. RAM [36] implemented the image classification task by adding an attention mechanism to an RNN. The self-attention mechanism proposed by Transformer in 2017 is different from the previous attention mechanism, which reduces the dependence on external information and focuses on the correlation between features. Ye et al. [37] proposed a cross-modal self-attention mechanism, in which the model can adaptively focus on important regions of the visual input. At the same time, a cross-frame self-attention module is introduced to improve the performance of video segmentation. Existing self-attention mechanisms mainly include spatial attention and channel attention, Fan et al. [38] adopted neural architecture search technology to achieve self-attention modeling in all dimensions. Wu et al. [39] proposed dimensional interactive self-attention mechanisms for feature extraction while reducing the computational load of the model and accelerating the training of the segmentation model. Fu et al. [40] proposed a channel and space dual self-attention mechanism and applied it to the semantic segmentation model. Although the self-attention mechanism can effectively guide the network to focus on important regions of the feature map, its computational complexity is high, which is not conducive to deployment at every layer of the network. To this end, we propose a PLAM module with low computational complexity in SMA-Net.

## 3. Method

In this section, we first propose the overall structure of SMA-Net. Then, the core modules of the network are introduced in detail, including edge feature fusion, the self-attentive channel attention mechanism and the spatial linear attention mechanism. Finally, the loss function used for training is described.

### 3.1. Network Structure

**Figure 1:**
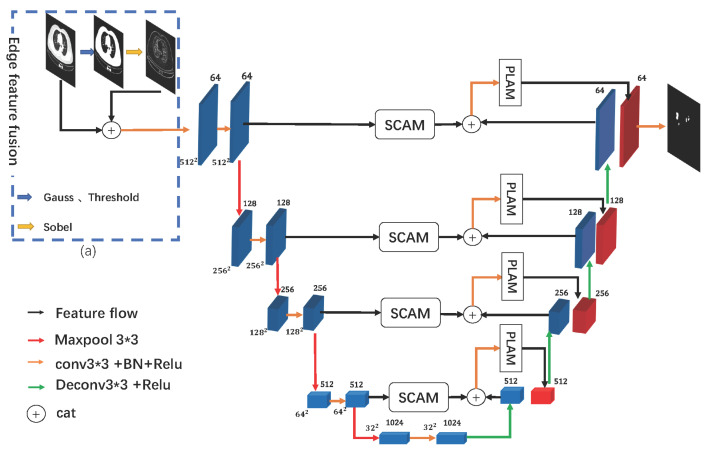
The network structure of SMA-Net. (**a**) The edge feature fusion in the blue dashed box. After the edge feature fusion is completed, the image is fed into the segmentation network. The segmentation network has four layers. Each layer has a corresponding channel attention mechanism and a spatial linear attention mechanism. The input image and the segmentation result output by the network have the same resolution.

The structure proposed for SMA-Net is shown in Figure 1. It can be seen that the original CT image is first fused with its corresponding edge features to obtain the input tensor of the network. Then, the input feature map is divided into two directions after the convolution and activation operations. The feature map is sent to the SCAM module, and it is also sent to the next layer by pooling for further feature extraction. SMA-Net performs downsampling four times for the features. The feature map is reduced from a resolution size of 512*512 to 32*32. Then, the feature map begins upsampling. After upsampling, the feature map is concatenated with the encoder feature map in the same layer after passing through the SCAM module, and then the obtained feature map is sent to the PLAM module to obtain a feature map with rich semantic information. The low-level features contain the contour semantic information of the lesion, and the high-level features contain the high-level semantic information of the lesion. After concatenating the low-level features and high-level features, the encoder obtains a lesion information-rich feature map. Next, the feature map is further upsampled, and then the above operation is repeated to upsample the feature map to the original image size. Finally, the channel is compressed to find the final segmentation result.

### 3.2. Edge Feature Fusion

In the semantic segmentation models applied to medical images, most of them use an encoding-decoding structure as the overall architecture. The encoder extracts the feature maps from the images through convolution and pooling operations. The low-level feature maps often contain more edge information on the lesions in the CT images. However, in the process of downsampling, the edge details in the feature map will be partially lost. To solve the loss of edge information, we propose fusing CT images with their edge features to add spatial detail information from the source of the model input. As shown in Figure 1a, we first perform a Gaussian filtering process on the CT image. The idea of Gaussian filtering is to suppress noise and retain detailed information by the weighted average of the pixels. Then, a thresholding process is carried out to obtain a binary map U:

(1) U=T(G(X,k=3),t=127)

where *G* denotes the Gaussian filtering operation, *k* is the filter size and *X* is the grayscale map of the input CT image, while *t* denotes the image thresholding and was set to 127 in this paper. Usually, if the value of *k* is set too large, it will cause the image to blur after Gaussian filtering. Therefore, *k* was set to 3 according to our experience. Considering that the background of the lung CT image is black, but the organs and lesions are white, *t* was set to 127 as the threshold of binarization. The Sobel operator was then used to calculate the gradient in the X and Y directions for the binary map, and the two gradients were combined to obtain the edge feature map. Finally, the model input Z was obtained by fusing the extracted edge feature map with its CT image:

(2) I=[S(U,g=x)+S(U,g=y)]

(3) Z=C(X,I)

where *S* denotes the Sobel operator operation, *g* denotes the directional gradient, *C* denotes the concatenation in the channel dimension and *x* and *y* represent the X and Y directions, respectively.

### 3.3. Self-Attentive Channel Attention Mechanism (SCAM)

To improve the semantic segmentation performance, U-shaped networks concatenate high-level features with low-level features to obtain richer semantic information. In the process of concatenation, a redundancy channel of the feature map often occurs. Therefore, channel attention modules (such as the classical SE module) are usually added to the network in order to emphasize the meaningful features of the channel axis. The SE module obtains the compressed feature vectors by global average pooling of the feature maps, and then the obtained compressed feature vectors go through the full connection layer to generate the weight of each channel of the feature map. The SE module is simple and easy to apply to the model. However, the global averaging pooling operation in the SE module results in a loss of semantic information.

**Figure 2:**
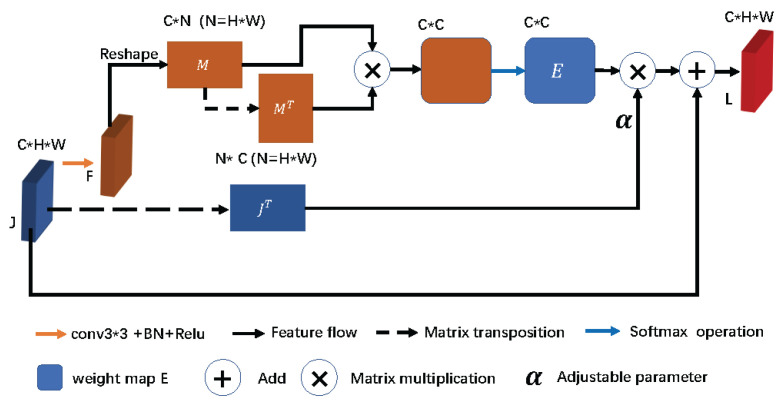
**SCAM**: self-attentive channel attention mechanism.

In order to solve this problem, we propose a self-attentive channel attention mechanism (SCAM) module, shown in Figure 2. Instead of compressing the feature map by global average pooling, the module first performs a convolution operation on the feature map *J* input to the SCAM module to obtain the feature map $F\in R^{C\times H\times W}$, shown in Equation (4). The feature map *F* is then reshaped to obtain the matrix $M\in R^{C\times N}$, and then the transposes of matrix *M* and *M* are calculated as the matrix product. Finally, using softmax to activate the matrix product yields the channel attention weight map *E*.

(4) F=f(J,k=3)

(5) $$E_{ji}=\frac{\exp\left(M_{i}\cdot M_{j}\right)}{\sum_{i=1}^{C}\exp\left(M_{i}\cdot M_{j}\right)}$$

where $E_{ji}$ denotes the effect of the *i*th channel on the *j*th channel. After obtaining the weight map *E*, *E* and the transpose of matrix *J* are calculated as matrix products. This assigns the values in the weight map to each channel of *J*. Given the idea of residual networks, the result of the product is multiplied by the adaptive coefficient α and then summed with *J* to obtain the final output *L* of the SCAM module:

(6) $$L=a\sum_{i=1}^{c}\left(E_{ji}J^{⊤}\right)+J$$

where the initial value of *a* is set to zero and can be changed with the needs of the network during the training process. *L* is used as the output of the input *J* passing through the SCAM module. *L* is then connected in series with its corresponding high-level features in the decoder.

### 3.4. Spatial Linear Attention Mechanism (PLAM)

**Figure 3:**
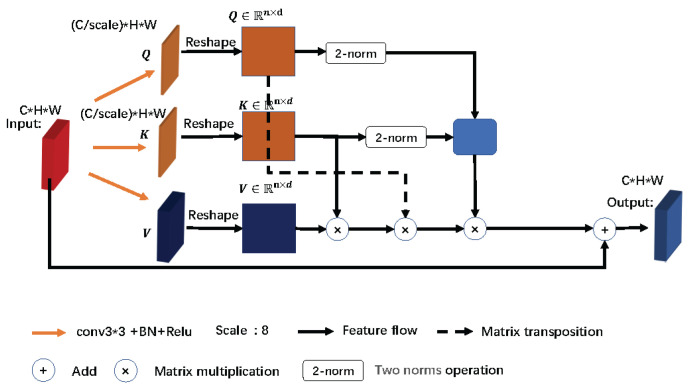
**PLAM**: Spatial linear attention mechanism structure.

After completing the concatenation of low-level features with high-level features, the decoder obtains a semantic-rich feature map. However, not all regions of this rich semantic information are equally important for lesion segmentation. To enhance the representation of key regions, we introduce the spatial linear attention module, shown in Figure 3. Before introducing the spatial linear attention module, we first review the principle of the compressed dot product attention mechanism (scaled-dot attention (SDA)), given in Equation (7):

(7) $$\mathrm{Attention}\;\left(Q,K,V\right)=\mathrm{softmax}\left(\frac{QK^{T}}{\sqrt{d_{k}}}\right)V$$

where *Q*, *K* and *V* denote the query matrix, the key matrix and the value matrix, respectively. These three matrices are obtained by convolving the input feature map through compressing the number of channels and then reshaping the feature map. $\sqrt{d_{k}}$ denotes the scaling factor. The overall dot product attention mechanism can be summarized as modeling the similarity between pixel points by matrix multiplication, and the softmax function is used to activate the matrix multiplication result.

However, since $Q\in R^{n\times d}$, $K\in R^{n\times d}$ and $V\in R^{n\times d}$, where n = W*H, W and H represent the width and height of the feature map, respectively, the complexity of the dot product attention mechanism is $O\left(n^{2}\right)$, which renders SDA limited by the image resolution. Moreover, the resolution of the CT images is usually large, and if SDA is used directly, it will exceed the computational power of the computer. If the resolution of the CT image is scaled, then much detailed information is lost in the image.

In order to improve SDA, we propose a spatial linear attention mechanism module. The complexity of the module is reduced from $O\left(n^{2}\right)$ to O(n), which allows the module to be flexibly applied to segmentation networks. We start by equivalently rewriting Equation (7) as Equation (8). Because PLAM does not use a scaling factor, $\sqrt{d_{k}}$ is removed from Equation (8). Equation (8) represents the result of the ith row of the matrix obtained from the feature map after feeding into the dot product attention mechanism:

(8) $$\;\mathrm{Attention}\;{\left(Q,K,V\right)}_{i}=\frac{\sum_{j=1}^{n}e^{q_{i}^{⊤}k_{j}}v_{j}}{\sum_{j=1}^{n}e^{q_{i}^{⊤}k_{j}}}$$

where $e^{q_{i}^{⊤}k_{j}}$ is essentially a weighted average over $v_{j}$, so Equation (8) can be generalized to a general form by replacing the softmax function with the general function as given in Equation (9):

(9) $$\;\mathrm{Attention}\;{\left(Q,K,V\right)}_{i}=\frac{\sum_{j=1}^{n}\mathrm{sim}\left(q_{i},k_{j}\right)v_{j}}{\sum_{j=1}^{n}\mathrm{sim}\left(q_{i},k_{j}\right)}$$

where $\mathrm{sim}\left(q_{i},k_{j}\right)\geq 0$. In order to reduce the complexity of Equation (9), the order of concatenation of $q_{i}$, $k_{j}$ and $v_{j}$ needs to be changed, and the normalization of $q_{i}$ and $k_{j}$ needs to be solved. In the construction of the linear attention mechanism, we start with Taylor expansion and turn $e^{q_{i}^{T}k_{j}}$ into $1+q_{i}^{T}k_{j}$:

(10) $$e^{q_{i}^{T}k_{j}}\approx 1+q_{i}^{T}k_{j}.$$

According to the Taylor expansion of Equation (9), $\mathrm{sim}\left(q_{i},k_{j}\right)=1+q_{i}^{T}k_{j}$. Since we need to normalize $q_{i}$ and $k_{j}$ and ensure that $\mathrm{sim}\left(q_{i},k_{j}\right)>0$, we can use the two norms of the matrix for normalization. Following this, Equation (8) can be equated to Equation (11):

(11) $$\;\mathrm{Attention}\;{\left(Q,K,V\right)}_{i}=\frac{\sum_{j=1}^{n}\left(1+{\left(\frac{q_{i}}{{∥q_{i}∥}_{2}}\right)}^{T}\left(\frac{k_{j}}{{∥k_{j}∥}_{2}}\right)\right)v_{j}}{\sum_{j=1}^{n}\left(1+{\left(\frac{q_{i}}{{∥q_{i}∥}_{2}}\right)}^{T}\left(\frac{k_{j}}{{∥k_{j}∥}_{2}}\right)\right)}$$

By modifying the original form of the attention mechanism, we have completed the construction of a spatial linear attention mechanism.

### 3.5. Loss Function

Due to the existence of small lesions in the CT images of COVID-19, the early clinical manifestations of COVID-19 are not obvious. The small lesion part of the CT images can be used as a basis for the early diagnosis of COVID-19. When the proportion of pixels in the target region is small, network training becomes more difficult, so small lesions are easily ignored in the network training process. Therefore, after the network has been built, it is important to choose a suitable loss function that is appropriate for the segmentation task. The Dice loss function, which is often used in segmentation tasks, cannot meet the segmentation needs of small lesions related to COVID-19. In order to fit the segmentation task, we chose the Tversky loss function, given in Equation (12):

(12) $$TL\left(\alpha ,\beta \right)=\frac{\sum_{i=1}^{N}p_{0i}g_{0i}}{\sum_{i=1}^{N}p_{0i}g_{0i}+\alpha \sum_{i=1}^{N}p_{0i}g_{1i}+\beta \sum_{i=1}^{N}p_{1i}g_{0i}}$$

where α and β are the parameters, which were set to 0.3 and 0.7, respectively, in this paper, while $p_{0i}$ represents the probability of a pixel point being diseased, $g_{0i}$ is one and $g_{1i}$ is zero when the pixel point is diseased and $p_{1i}$ represents the probability of a pixel point being non-diseased. When the pixel point is non-lesioned, $g_{0i}$ is zero, and $g_{1i}$ is one. As can be seen from Equation (12), the trade-off between false negatives and false positives can be controlled when adjusting the values of α and β. The value of β is taken to be 0.7 greater than α, improving the sensitivity by emphasizing false negatives. This allows the network to focus on small lesion areas during training, thus addressing the problem of data imbalance in CT images of patients with COVID-19.

## 4. Experiment

### 4.1. Data and Preprocessing

Regarding the public COVID-19 segmentation dataset, the public dataset used in this paper is from zendo [41]. The dataset contains 20 COVID-19 CT scans, including lung and lesion segmentation labels. The dataset was annotated by two radiologists and examined by an experienced radiologist. In this study, 2237 CT images were selected for the experiment. To speed up the convergence of the network and improve efficiency, some preprocessing operations were performed on this dataset. We cropped the CT images to a resolution of 512*512 size to reduce the amount of calculation in the training process. The CT images were then normalized. Image normalization is the process of centering the data, which can improve the generalization of the network.

### 4.2. Experimental Set-Up

For the baseline, in the lesion segmentation experiments, our proposed SMA-Net was compared with the classical networks UNet, UNet++ and VUNet. In addition, we also referred to the advanced semantic segmentation networks Deeplabv3, FCN, PSP and SegNet. Moreover, we also compared three newly proposed COVID-19 lesion segmentation networks: AnamNet, JCS and Inf-Net.

**AnamNet** [30]: A lightweight CNN based on deformation depth embedding for a segmentation network of COVID-19 chest CT image anomalies which can be deployed to mobile terminals.

**JCS** [42]: A novel combined classification and segmentation system for real-time and interpreted COVID-19 chest CT diagnosis.

**Inf-Net** [31]: A semi-supervised segmentation framework based on a random selection propagation strategy for a network with a fully supervised form, which we selected for its fully supervised approach.

### 4.3. Evaluation Indicators

We evaluated the similarity of the SMA-Net segmentation results and labels using the DSC metric and the segmentation accuracy of SMA-Net using the overlap intersection ratio (IOU). We used the sensitivity (SEN) measure to evaluate the ability of SMA-Net to identify the focal regions in CT images. We used the specificity (SPE) measure to evaluate the ability of SMA-Net to identify healthy regions in CT images. The formulae for the evaluation of the indicators are as follows:

(13) $$\mathrm{DSC}=\frac{2\mathrm{TP}}{2\mathrm{TP}+\mathrm{FP}+\mathrm{FN}}$$

(14) $$\mathrm{IOU}=\frac{\mathrm{TP}}{\mathrm{TP}+\mathrm{FP}+\mathrm{FN}}$$

(15) $$\mathrm{SEN}=\frac{\mathrm{TP}}{\mathrm{TP}+\mathrm{FN}}$$

(16) $$\mathrm{SPE}=\frac{\mathrm{TN}}{\mathrm{TN}+\mathrm{FP}}$$

where the TP value represents the number of pixels in the CT lesion region that the network segmented accurately, FP represents the number of pixels in the lesion region that the network failed to segment in the CT image, TN represents the number of pixels in the healthy region that the network segmented correctly and FN represents the number of pixels in the healthy region that the network failed to segment correctly in the CT image.

### 4.4. Segmentation Results

To compare the segmentation performance of SMA-Net, we refer to the classical medical image segmentation network UNet and its variant UNet++. In addition, we also refer to the advanced semantic segmentation networks Deeplabv3, FCN and SegNet. For the three recently proposed COVID-19 lesion segmentation networks (AnamaNet, JCS and Inf-Net), we also conducted comparative experiments. The quantitative results are shown in Table 2. It can be seen that for the other methods, our proposed SMA-Net achieved a significant improvement in the IOU metric, with a 7.8% improvement compared with UNet. The DSC coefficient also achieved its best result. We attribute this improvement to our edge feature fusion module as well as the self-attentive channel attention mechanism and spatial linear attention mechanism. Thanks to the two attention mechanisms guiding SMA-Net, SMA-Net can sample a richer feature map of semantic information during feature extraction.

**Figure 4:**
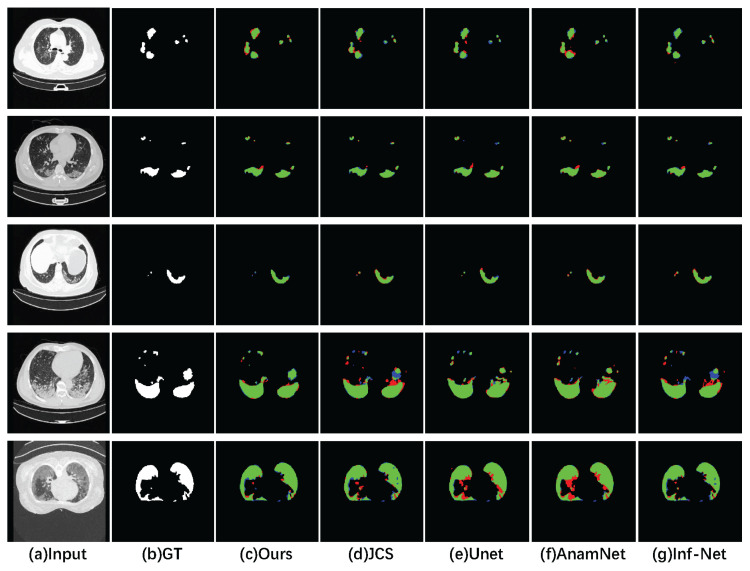
Visual comparison of lesion segmentation results using different networks. (**a**) CT images. (**b**) Ground truth. (**c**,**d**,**e**,**f**,**g**) Segmentation results for SMA-Net, JCS, UNet, AnamNet and Inf-Net, respectively. The green, blue and red regions refer to true positive, false negative and false positive pixels, respectively.

Figure 4 shows a visual comparison of SMA-Net with UNet and its two variants, VUNet and UNet++, with three newly proposed COVID-19 lesion segmentation networks (AnamaNet, JCS and Inf-Net).The green, blue and red regions refer to true positive, false negative and false positive pixels, respectively. It can be seen that SMA-Net was closest to the ground truth. In contrast, many false positive pixels appeared in the UNet and AnamNet segmentation results. Due to our choice of the Tversky loss function, SMA-Net achieved good results in the segmentation of small lesions. Compared with the other networks, our increased sensitivity of the loss function to small lesion regions allowed the network to segment the small lesion regions well.

### 4.5. Ablation Learning

**Figure 5:**
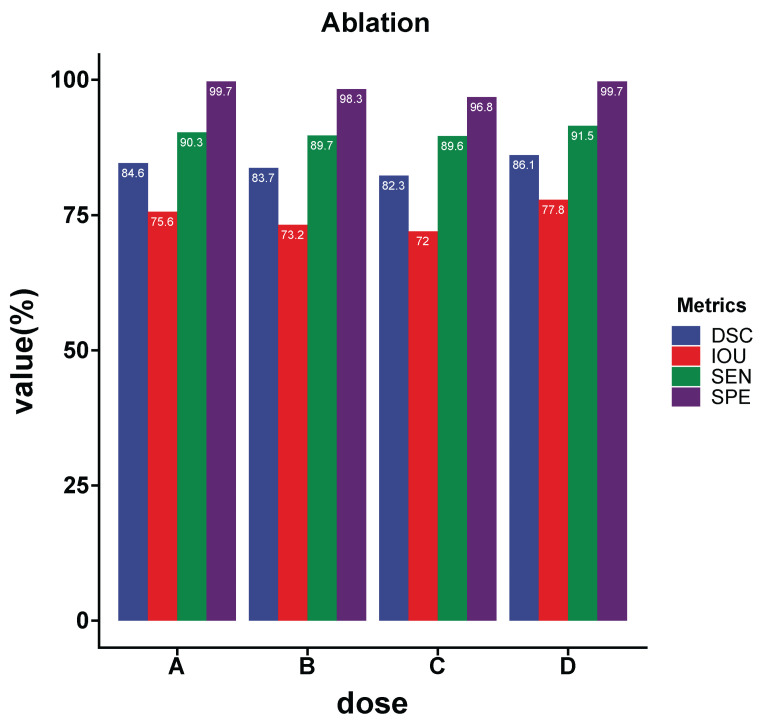
Results of the ablation experiment. (**A**) SMA-Net without SCAM. (**B**) SMA-Net without PLAM. (**C**) SMA-Net without the feature fusion. (**D**) SMA-Net.

In this section, we experimentally demonstrate the performance of key components of SMA-Net, including the edge feature fusing module, the self-attention channel attention mechanism module (SCAM) and the spatial linear attention mechanism module (PLAM). In Figure 5, A is the SMA-Net without the SCAM module, B is the SMA-Net without the PLAM module, C is the SMA-Net with the edge feature fusion module removed, and D is the complete SMA-Net.

- Effectiveness of **SCAM**: To explore the SMA-Net’s self-attentive channel attention module, we propose two benchmarks, shown in Figure 5: A (SMA-Net without SCAM) and D (SMA-Net). The results show that SCAM was effective at improving network performance.
- Effectiveness of **PLAM**: From Figure 5, it can be observed that the IOU values decreased more with B (SMA-Net without PLAM) compared with D. This indicates that the spatial linear attention mechanism has an important role in guiding the network to learn to segment the lesion area, allowing SMA-Net to focus more on the pixels in the lesion area.
- Effectiveness of edge feature stitching: After the fusing of edge features is completed, the encoder obtains richer semantic information. As can be seen from Figure 5, C had the lowest IOU metric compared with A, B and D, which indicates that edge features are important for the detail complement of CT images.

### 4.6. Selection of the Loss Function

After the construction of SMA-Net was completed, the selection of the loss function had a great impact on the performance of the network. Therefore, for different semantic segmentation tasks, the selection of the loss function was based on the characteristics of the task. Commonly used loss functions include the Dice loss (DL) function, balanced cross-entropy loss function (BCE) for binary classification tasks and the weighted cross-entropy loss function (WCE). In addition, we also selected excellent loss functions that have been used for semantic segmentation in recent years, namely asymmetric loss functions (AL), TverskyLoss (TL) and PenaltyGDiceLoss (PL):

(1) Asymmetric loss functions (AL): A novel loss function is designed to address the problem of positive and negative sample imbalance in classification tasks. Adaptive methods are proposed to control the asymmetric rank.

(2) TverskyLoss (TL): In order to solve the problem of data imbalance, a new loss function is proposed to improve the sensitivity of small lesion areas by adjusting the parameters of the Tversky index.

(3) PenaltyGDiceLoss(PL): This improves network segmentation performance by adding false negative and false positive penalty terms to the generalized dice coefficients (GD).

### 4.7. Comparison of Loss Functions

**Figure 6:**
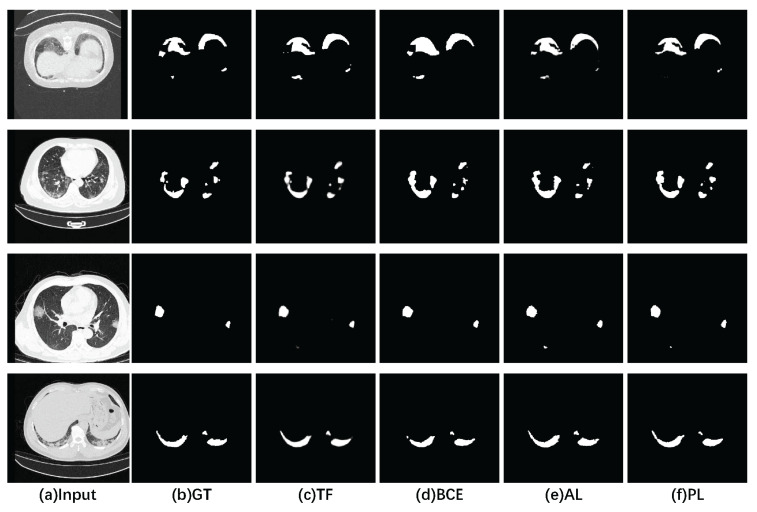
Visualization comparison of SMA-Net lesion segmentation with different loss functions. (**a**) CT images. (**b**) Ground truth. (**c**,**d**,**e**,**f**) Segmentation results of SMA-Net using TL, BCE, AL and PL loss functions, respectively.

As can be seen from Table 3, TverskyLoss (TL) performed the best among the three indicators of IOU, DSC and SPE. Compared with the BCE loss function, the IOU and DSC coefficients improved by 6.8% and 7%, respectively. Among them, AL performed the best in terms of sensitivity. We also made a visual comparison of the output results of SMA-Net with different loss functions. As shown in Figure 6, the results from TL were more sensitive for small lesion regions and could do well in segmenting small lesions. In contrast, the lack of segmentation for small lesion regions can be observed in the segmentation results of BCE as well as AL.

### 4.8. Sensitivity to Parameters

There are two parameters, α and β, in Equation (12). In order to understand their impact on model performance, we employed a grid search means to carry on the parameter sensitivity analysis. The range of values of α and β was (0, 1), and we set α + β = 1, where α and β control the weights of the false negatives and false positives, respectively. By turning up the β value to emphasize the false negatives, the model focused more on the false negatives. Table 4 shows that the higher the value of β, the higher the sensitivity (SEN). The specificity (SPE) was highest when both α and β were 0.5. The model achieved a good balance between sensitivity and specificity when α = 0.3 and β = 0.7, and the model segmentation performed best.

## 5. Discussion

The experimental dataset we used contains CT images of patients with severe diseases and CT images of patients with minor diseases. The segmentation results of both CT images fed into SMA-Net outperformed the other networks. Because SMA-Net is an end-to-end segmentation network, the stability of SMA-Net is good under complexity. Through experimentation, we found that SMA-Net had excellent segmentation performance. However, SMA-Net had some weaknesses compared with other networks. The segmentation accuracy of SMA-Net was higher than AnamNet, but the computational power required for SMA-Net is higher than AnamNet. JCS can perform classification tasks while implementing segmentation, while SMA-Net does not yet have classification capabilities. The semi-supervised version of the Inf-Net training process relies on only a small number of labels, while the SMA-Net training process requires all labels. The implementation challenge is mainly the high computing power required by our proposed SMA-Net. We experimented with a 2080 Ti graphics card. Because hospital facilities may not have high-calculus graphics cards at the time of deployment, the challenge we face is to improve the complexity of the network without compromising the performance of the network segmentation.

## 6. Conclusions

To improve the efficiency of diagnosis of COVID-19, we developed a COVID-19 lesion segmentation network. In our network, we propose an edge feature fusion module which allows the network to capture more edge feature information. In addition, we introduce a self-attentive channel attention mechanism and a spatial linear attention mechanism to improve the network performance. Two attention mechanisms guide SMA-Net, which captures lesion areas more accurately during feature extraction. Compared with the classical medical image segmentation network UNet, the DSC and IOU of SMA-Net were improved by 7% and 7.8%, respectively. Although our method achieved good results in terms of performance, it still has the following shortcomings: (1) the network has high computational complexity, and (2) the network does not perform the classification task simultaneously. Therefore, our future work will try to start from light weighting of the model, considering the use of expanded convolution instead of the original convolution operation and using pruning methods to compress the network parameters. The network is realized to be lightweight and easy to implement. In the future, we will also try to integrate the fully connected layer into the network and achieve simultaneous network classification and segmentation to improve the diagnosis of COVID-19.

## Figures and Tables

**Table 2:** Comparison of lesion segmentation performance.

Methods	DSC	IOU	SEN	SPE
UNet	0.797	0.701	0.869	**0.998**
UNet++	0.754	0.687	0.836	0.993
VUNet	0.813	0.711	0.879	0.968
Deeplabv3	0.773	0.646	0.861	0.997
FCN	0.689	0.612	0.795	0.876
PSP	0.656	0.589	0.768	0.858
Segnet	0.731	0.634	0.854	0.993
AnamNet	0.808	0.71	0.846	0.979
JCS	0.847	0.754	0.852	0.989
Inf-Net	0.818	0.723	0.871	0.985
**Ours**	**0.861**	**0.778**	**0.915**	0.997

**Table 3:** Comparison of SMA-Net results under different loss functions.

Loss	DSC	IOU	SPE	SEN
**BCE**	0.834	0.743	0.979	0.896
**WCE**	0.823	0.726	0.983	0.852
**DC**	0.783	0.697	0.997	0.884
**GD**	0.799	0.689	0.973	0.854
**AL**	0.824	0.727	**0.998**	0.897
**TL**	**0.861**	**0.778**	0.997	**0.915**

**Table 4:** Performance metrics for different values of parameters α and β used in training SMA-Net.

Parameters	DSC	IOU	SEN	SPE
**a = 0.1, b = 0.9**	0.761	0.643	**0.933**	0.997
**a = 0.2, b = 0.8**	0.821	0.724	0.919	0.983
**a = 0.3, b = 0.7**	**0.861**	**0.778**	0.915	0.997
**a = 0.4, b = 0.6**	0.838	0.741	0.903	0.997
**a = 0.5, b = 0.5**	0.826	0.715	0.878	**0.998**

## Data Availability

This research does not create new data.
